# Environmental determinants of cerebral haemorrhage in older adults: behavioural pathways and population health implications

**DOI:** 10.3389/fpubh.2025.1670520

**Published:** 2025-12-23

**Authors:** Qiang Ji, Yawei Hou

**Affiliations:** 1 Institute of Chinese Medical Literature and Culture, Shandong University of Traditional Chinese Medicine, Jinan, China

**Keywords:** intracerebral haemorrhage, environmental exposures, older adults, fine particulate matter, behavioural pathways, air-quality regulation

## Abstract

Intracerebral haemorrhage (ICH) is a rapidly fatal cerebrovascular catastrophe that claims a disproportionate share of stroke deaths among older adults despite decades of progress in acute care. Emerging research now implicates a constellation of non-biomedical contextual stressors ambient fine particulate matter, traffic-derived gases and noise, thermal volatility, and bio-accumulative heavy metals, themselves patterned by social determinants of health and political-commercial decision-making as pivotal but still under-recognised drivers of small-vessel rupture. This review synthesises epidemiological, behavioural and translational evidence to illuminate how pollutant-driven sympathetic arousal, sleep fragmentation, physical inactivity and impaired thermoregulation converge on age-accentuated endothelial fragility, thereby lowering the haemodynamic threshold for cerebral bleeding. We further map the geo-temporal and socio-spatial inequities in these exposures rooted in social, political and commercial determinants of health that tether disadvantaged communities to higher exposure loads and outline the corresponding gradients in ICH incidence, mortality and disability-adjusted life-years. We appraise the preventive leverage of integrated structural policies that decarbonise urban transport, regulate commercial determinants, dampen nocturnal noise, expand equitable green infrastructure and fortify climate resilience. By advancing a behavioural–environmental framework that links modifiable exposures to actionable pathways, this article furnishes clinicians, public-health practitioners and policymakers with a coherent agenda for mitigating the impending surge of environmentally mediated cerebral haemorrhage in ageing societies.

## 1 Introduction

Intracerebral haemorrhage (ICH) represents the most lethal subtype of stroke, accounting for roughly 10–15% of all cerebrovascular events yet contributing disproportionately to stroke-related mortality and long-term disability in ageing societies (1–3). Global epidemiological analyses conducted within the Global Burden of Disease framework indicate that absolute ICH incidence has risen steadily over the past three decades, primarily because the population aged ≥65 years—the demographic at highest risk—continues to expand worldwide (4–6). Despite advances in acute neurocritical care, case-fatality remains close to 40% at 1 month and functional dependence is frequent among survivors (7–9). These observations underscore the urgency of identifying modifiable upstream factors that can be targeted before haemorrhage occurs.

Beyond established medical determinants such as hypertension, cerebral amyloid angiopathy and antithrombotic use, a growing body of evidence implicates a spectrum of environmental exposures in the aetiology and precipitants of ICH. Cohort and case-crossover studies conducted in North America, Europe and East Asia consistently associate short-term peaks in ambient fine particulate matter (PM₂.₅) with an elevated risk of haemorrhagic stroke admissions, particularly among older adults whose vascular resiliency is already compromised (10–12). Similarly, chronic exposure to traffic-related nitrogen oxides, residential proximity to major roadways, and incremental increases in household or community noise levels have each been linked to higher ICH incidence or fatality in multicentre investigations (13, 14). Extreme temperatures—both heat waves and cold spells—further exacerbate cerebrovascular fragility by inducing rapid oscillations in blood pressure and coagulability, phenomena shown to precede spontaneous haemorrhage in susceptible elders (15, 16). Heavy metals such as lead and cadmium, often co-distributed with ambient particulate mixtures, have also been implicated through biomonitoring studies demonstrating dose-dependent associations with cerebral microbleeds and small-vessel pathology (17, 18).

Environmental determinants rarely act in isolation; rather, they converge on behavioural and physiological pathways that magnify cerebrovascular vulnerability across the lifespan. Air pollution and excessive neighbourhood noise, for example, diminish outdoor physical activity, disrupt sleep architecture and promote chronic sympathetic activation—behavioural cascades that sustain hypertension and endothelial dysfunction, recognised proximate triggers of ICH (19–21). Conversely, constrained access to green space or walkable environments may foster sedentary lifestyles and central obesity, further heightening haemorrhagic risk in later life (22, 23). Socio-spatial inequities as expressions of broader social determinants of health and of political and commercial choices about land use, transport and energy frequently dictate the distribution of these hazards: older adults residing in socio-economically deprived districts are disproportionately exposed to higher pollutant loads, insufficient urban cooling infrastructure and sub-standard housing, thereby compounding age-related cerebrovascular degeneration (24, 25). Framing these patterns as social, political and commercial determinants of health clarifies that prolonged exposure to hazardous environments is not an inevitable accompaniment of ageing but the consequence of modifiable policy and market decisions made across space and time.

The population-level consequences are substantial. Comparative risk assessments attribute up to one quarter of all stroke deaths in low- and middle-income settings to jointly modifiable environmental and behavioural factors, with the haemorrhagic subtype contributing a sizeable share of this preventable burden (26–28). Moreover, the intersection of climate change, urbanisation and demographic ageing is poised to intensify exposure–outcome gradients unless mitigation strategies are enacted.

Against this backdrop, the present review synthesises contemporary evidence on environmental and broader social, political and commercial determinants of cerebral haemorrhage in older adults, delineates behavioural pathways through which these external stressors translate into cerebrovascular injury, and examines resultant patterns of disease burden and disparity at the population level. By systematically integrating findings from epidemiology, environmental health and social neuroscience, we aim to furnish a coherent framework for prevention and policy while highlighting priorities for future interdisciplinary research.

## 2 Environmental exposures influencing intracerebral haemorrhage in ageing populations

The ecological milieu in which older adults age exerts quantifiable pressure on the integrity of small cerebral vessels. A converging body of population-based evidence implicates several external stressors—most prominently combustion-derived aerosols, traffic-related gases and noise, thermal extremes, and ubiquitous toxic metals—in precipitating intracerebral haemorrhage (ICH) over and above classical medical determinants. Table 1 synthesises these exposures, their principal pathophysiological correlates, and the host features that amplify vulnerability in late life.

**Table 1:** Descriptive summary of major environmental exposures linked to intracerebral haemorrhage in older adults.

Environmental factor	Principal cerebrovascular mechanisms	Age-related amplifiers
Fine particulate matter (PM₂.₅)	Endothelial inflammation, vasoconstriction, oxidative DNA damage, blood–brain barrier disruption	Accumulated vascular stiffening, diminished antioxidant reserves
Nitrogen dioxide and other traffic-derived gases	Cerebral arteriolar dysfunction, dysregulation of coagulation cascade, sympathetic activation	Higher baseline pulse-pressure variability, polypharmacy with vasoactive agents
Road-traffic and community noise	Sleep fragmentation, chronic cortisol elevation, surges in nocturnal blood pressure	Increased prevalence of insomnia, impaired baroreflex sensitivity
Extreme ambient temperature (heat waves, cold spells)	Rapid haemodynamic oscillations, haemoconcentration, altered platelet reactivity	Thermoregulatory inefficiency, reduced mobility limiting behavioural adaptation
Heavy metals (lead, cadmium) co-distributed with particulate mixtures	Microangiopathic degeneration, promotion of cerebral microbleeds, interference with calcium-dependent vascular signalling	Greater cumulative body burden from historical exposure, osteoporotic mobilisation of skeletal lead stores

Long-term inhalation of fine particulate matter remains the most consistently documented environmental determinant of ICH. Among U. S. Medicare beneficiaries, each 5 μg m^−3^ increment in annual PM₂.₅ concentration corresponded to an 11% increase in intracranial bleeding admissions (29, 30). Global burden assessments have further attributed a substantial fraction of haemorrhagic stroke mortality to sustained exposure levels well below current regulatory thresholds (31, 32). Mechanistically, sustained particulate inhalation promotes arteriolar lipohyalinosis and impairs cerebral autoregulation—alterations that synergise with age-associated hypertension to lower the pressure at which vessel rupture ensues.

Traffic-related gaseous pollutants exert independent and joint effects beyond particulate co-pollutants. In a prospective cohort of post-menopausal women, daily nitrogen-dioxide exposures were associated with a 21% rise in incident haemorrhagic stroke after multivariable adjustment (33, 34). Multipollutant analyses that incorporated concurrent road-traffic noise demonstrated supra-additive risk elevations for cerebrovascular events, underscoring the intertwined nature of chemical and acoustic stressors in urban corridors (35, 36).

Meteorological stress also holds particular relevance for ageing cerebrovasculature. Warm-season cold spells and summer heat anomalies each precipitated sharp spikes in haemorrhagic stroke admissions across multi-city time-series, with the greatest excess risk observed in individuals aged ≥75 years (37–39). Parallel analyses of compound hot extremes corroborated these findings and suggested impaired heat dissipation and dehydration as proximate triggers (40, 41).

Persistent acoustic stimulation represents an under-recognised vascular insult. Danish registry studies reported that long-term residential road-traffic noise exceeding 65 dB(A) was positively associated with hospitalisations for ICH, particularly in neighbourhoods with low greenness where restorative buffers are scarce (42, 43). Experimental models indicate that nocturnal noise amplifies sympathetic tone and endothelial oxidative stress, effects that are magnified in senescent vessels.

Toxic metal contaminants embedded in airborne and dietary matrices appear to accelerate cerebral small-vessel degeneration. Systematic reviews link higher blood or urinary cadmium to elevated cerebrovascular mortality, including haemorrhagic subtypes (44, 45), while burden-of-disease modelling attributes a growing share of stroke deaths to cumulative lead exposure, with pronounced impacts in low-resource settings (46, 47). These metals accumulate over decades and may be remobilised from bone in osteoporosis, rendering older adults uniquely susceptible.

Mounting epidemiological and mechanistic data indicate that ambient aerosols, traffic-derived gases, persistent acoustic stress, thermal extremes, and bio-accumulative metals collectively shape a modifiable exposome that accelerates small-vessel injury and precipitates intracerebral haemorrhage in later life. These external stressors intersect with age-related haemodynamic instability, impaired endothelial repair, and sympathetic over-activation, thereby lowering the threshold for vessel rupture even in the absence of overt hypertension or amyloid angiopathy. Recognising this convergence clarifies the preventive potential of policies that curb combustion emissions, attenuate urban noise, and mitigate climate-related thermal volatility, while simultaneously guiding clinicians towards heightened surveillance of high-exposure subgroups.

## 3 Behavioural pathways translating environmental stressors into cerebrovascular vulnerability

Environmental stressors shape intracerebral haemorrhage susceptibility by modifying behaviours that govern haemodynamic stability, vascular metabolism and endothelial repair across late life (48, 49). Air- and noise-borne pollutants are prominent in this regard: multiyear device-based monitoring of more than 1 million nights of sleep demonstrated that incremental increases in PM₂.₅, NO₂ and carbon monoxide prolong total sleep time yet truncate deep-sleep duration, indicating poorer sleep quality and heightened nocturnal sympathetic tone in adults aged ≥60 years (50, 51). Parallel pooled analyses of 16 million participants show that every 10 dB(A) rise in long-term road-traffic noise elevates stroke incidence by 4% and stroke mortality by 3%, implicating chronic acoustic arousals, cortisol surges and sleep fragmentation in the cerebrovascular cascade (52, 53). Such dysregulated sleep and circadian instability accelerate overnight blood-pressure variability and impair endothelial nitric-oxide signalling—conditions that lower the threshold for vessel rupture in ageing perforating arteries.

Autonomic imbalance constitutes a second conduit linking environmental adversity to haemorrhagic risk. A meta-analysis of 33 panel studies found that a 10 μg m^−3^ increase in short-term PM₂.₅ exposure reduced time-domain and frequency-domain heart-rate-variability indices by up to 2.2%, signifying diminished parasympathetic modulation and persistent sympathetic activation (54, 55). Experimental and epidemiological work further indicates that nocturnal traffic noise provokes vascular oxidative stress and catecholaminergic release, effects that are amplified in older vessels with pre-existing stiffness (56–58). Sustained sympathetic over-drive augments pulse-pressure oscillations and promotes lipohyalinotic degeneration of small cerebral arteries, thereby facilitating intracranial bleed when blood pressure suddenly spikes.

Environmental conditions also sculpt daily activity patterns and metabolic load. Comprehensive review of cohort and experimental data reveals that high particulate episodes discourage outdoor physical activity and increase sedentary time among adults over 65, blunting the antihypertensive and weight-modifying benefits of exercise (59–61). Conversely, prospective follow-up of Chinese octogenarians showed that residence in high-greenness areas conferred a 40% reduction in incident hypertension, plausibly through facilitated walking, psychosocial stress relief and improved local air quality (29, 62). The resultant divergence in long-term blood-pressure trajectories partially explains why older adults in vegetation-sparse, high-pollution corridors experience disproportionate haemorrhagic stroke burdens.

Thermal extremes impose additional behavioural and physiological burdens. Reviews focusing on populations above 65 years document that heat stress elevates cardiovascular hospitalisations and mortality through dehydration-induced haemoconcentration, reduced plasma volume and compensatory surges in sympathetic activity; these responses are accentuated when high ambient temperatures coincide with elevated PM₂.₅, creating synergistic insults to vascular integrity (63–65). Adaptive behaviours—such as increased indoor confinement, air-conditioning use and altered fluid intake—may mitigate or inadvertently magnify these haemodynamic stresses, particularly in settings of limited cooling infrastructure.

Disrupted sleep architecture, chronic autonomic arousal, pollution-driven physical inactivity and maladaptive responses to thermal stress operate in concert to erode cerebrovascular resilience in later life. Targeting these modifiable behavioural pathways offers a pragmatic complement to traditional risk-factor control for preventing intracerebral haemorrhage in ageing populations.

## 4 Population-level patterns, disparities, and burden

Age-standardised intracerebral haemorrhage (ICH) incidence has declined modestly in many high-income jurisdictions over the past three decades; nevertheless, the absolute number of cases continues to rise because population ageing outpaces these epidemiological gains (66, 67). Global Burden of Disease analyses estimated more than 3 million incident ICH events and over 65 million disability-adjusted life-years (DALYs) in 2019, with adults ≥ 70 years accounting for nearly two-thirds of the total burden (68, 69). Region-specific projections for Europe illustrate the demographic shift: individuals aged ≥80 years generated 40% of ICH cases in 2019, a proportion forecast to exceed 60% by 2050 even under conservative ageing scenarios (70, 71).

Marked geographic and socioeconomic gradients persist. More than 80% of incident ICH and 90% of related DALYs now occur in low- and middle-income countries, where case-fatality remains high and post-stroke support infrastructure is limited (72, 73). Within high-income settings, census-tract analyses repeatedly demonstrate higher ICH incidence in neighbourhoods characterised by lower median income, greater material deprivation and a higher concentration of racialised minorities; multilevel modelling attributes up to 25% of this excess risk to contextual disadvantage manifest social determinants of health shaped by long-standing political and commercial disinvestment independent of individual vascular risk factors (74–76). Sex-stratified meta-analyses indicate that women experience lower age-specific incidence but worse functional outcomes, a disparity amplified in the very-old age stratum where dependency after haemorrhage intersects with caregiver scarcity.

Environmental determinants magnify these inequities through spatially patterned exposure profiles. Ambient fine particulate matter (PM₂.₅) concentrations exceed guideline levels in many rapidly urbanising regions, and short-term elevations have been associated with 7–15% increases in ICH admissions among older adults in case-crossover studies (50, 77, 78). Some study comparative risk assessment attributed roughly 18% of all stroke deaths in low-income settings to ambient air pollution, with haemorrhagic subtypes contributing a disproportionate share because of the heightened vulnerability of small cerebral vessels to oxidative stress and pressure surges (79, 80). Extreme heat and cold events further compound risk: quasi-Poisson time-series analyses in populations aged ≥65 years report 10–20% excess cerebrovascular mortality during heatwaves and winter cold spells, effects that are more pronounced for haemorrhagic than ischaemic stroke (26, 81).

**Figure 1:**
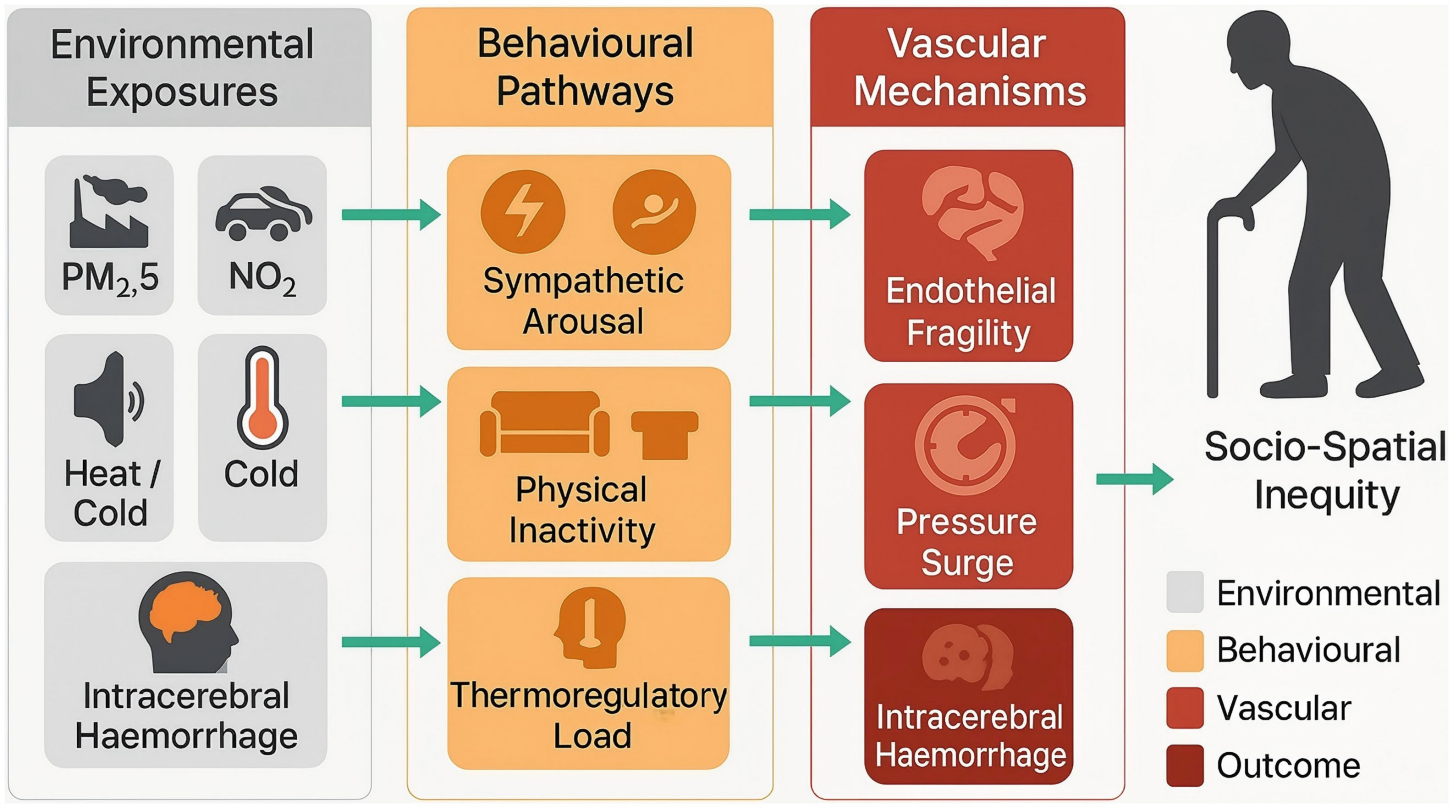
Integrated environmental–behavioural model of intracerebral haemorrhage (ICH) in older adults.

As shown in Figure 1, when exposures are combined, their population-attributable impact is substantial. Multipollutant burden modelling suggests that the joint contribution of PM₂.₅, traffic-related nitrogen oxides, non-optimal temperature, and persistent urban noise accounts for approximately one quarter of haemorrhagic stroke DALYs in East and South-East Asia, with synergistic amplification observed in megacities experiencing concurrent heat-air pollution episodes (8, 23). Socio-environmental clustering across space and time means that older residents in deprived districts shoulder both higher toxin loads and greater baseline comorbidity, resulting in steep exposure response gradients that are not evident in aggregate national statistics and that reflect enduring social-determinant inequalities.

Forward-looking scenario analyses incorporating demographic ageing, urbanisation trajectories and climate-change-associated thermal volatility predict a 30–40% increase in ICH events attributable to environmental factors between 2020 and 2050 if current emission and urban-heat-island trends persist. The projected economic costs—encompassing acute care, long-term institutionalisation and informal caregiving—are expected to rise disproportionately, given the concentration of burden in the very-old, who exhibit the highest case-fatality and disability rates. These forecasts underscore the necessity of integrating environmental mitigation with classical vascular risk-factor control to curb the impending escalation of haemorrhagic stroke burden in ageing societies.

## 5 Prevention, policy, and future research directions

Primary prevention of intracerebral haemorrhage in later life hinges on upstream reduction of the environmental stressors that destabilise small cerebral vessels. Because these exposures arise from zoning laws, transport and energy policies, and the commercial strategies of powerful industries, they are best conceptualised as social, political and commercial determinants of health rather than as immutable background risks. Cohort simulations indicate that attaining annual PM₂.₅ concentrations <5 μg m^−3^ would avert a substantive fraction of haemorrhagic strokes among adults ≥65 years, even in regions where conventional guideline values are already met (27, 82, 83). Convergent evidence from multipollutant analyses shows parallel benefits when nitrogen-dioxide peaks are dampened via traffic electrification and low-emission zones, and when night-time road-traffic noise is curtailed through speed regulation and acoustic barriers (22, 58, 84, 85). Climate-responsive urban greening, by lowering ambient temperatures and diluting particle and noise intensities, further extends vascular protection while introducing co-benefits for cardiometabolic fitness (23, 59, 86, 87). Collectively, these findings support an integrated source-control framework that aligns air-quality, noise-abatement, and climate-adaptation agendas to lower haemorrhagic stroke risk in ageing societies.

Behavioural mediation offers an additional, rapidly actionable layer of defence. Prospective data demonstrate that sustained exposure to clean air restores outdoor physical activity patterns and attenuates pollution-induced blood-pressure lability, effects particularly pronounced in older adults with limited baseline mobility (12, 48). Residential greenness and accessible cooling refuges mitigate heat- and noise-related sleep fragmentation, thereby reducing nocturnal sympathetic surges that precipitate vessel rupture (41, 45). Embedding environmental metrics into clinical counselling—e.g., advising older patients to synchronise exercise with real-time air-quality forecasts or to adopt portable air filtration during extreme events—may therefore amplify the efficacy of traditional vascular-risk management.

Regulatory policy must explicitly address the disproportionate exposure burdens borne by socio-economically deprived and racially minoritised communities, where older residents experience cumulative pollutant loads and constrained health-promoting resources as the downstream expression of adverse social determinants of health and structural racism (24, 79). Health-in-all-policies legislation that ties land-use approvals, housing standards, and transport funding to quantified cerebrovascular-health indicators would operationalise this equity mandate. Parallel incorporation of haemorrhagic-stroke endpoints into cost–benefit analyses for climate-mitigation and energy-transition initiatives is warranted, given projection models attributing a sizeable share of future ICH growth to heat–pollution co-occurrence under high-emission scenarios (23, 29).

Research priorities centre on elucidating exposomic synergies and causal pathways specific to senescent cerebrovasculature, with explicit attention to how these risks vary across geo-temporal and socio-economic contexts. Large-scale panel studies integrating personal multisensor pollution, temperature, and noise monitoring with ambulatory blood-pressure and sleep metrics are needed to refine short-lag exposure–response functions. Longitudinal cohorts that combine high-resolution environmental data with neuro-imaging of cerebral microbleeds will clarify dose thresholds for irreversible small-vessel injury (40, 56). Rigorous interdisciplinary spatial-epidemiological designs that apply geostatistical methods such as geographically weighted regression (GWR) and multiscale geographically weighted regression (MGWR) can quantify how the strength of associations between environmental exposures, social determinants and ICH outcomes varies across neighbourhoods and over time, thereby informing locally tailored interventions. Natural-experiment evaluations of emission-control, greening, or insulation interventions should routinely stratify outcomes by age, sex, and socioeconomic status to inform targeted deployment. Translational work linking environmental modification to laboratory markers of endothelial repair and cerebrovascular stiffness in older adults will facilitate mechanistic convergence between epidemiology and vascular biology (9, 53).

The converging epidemiological, clinical, and policy evidence base underscores that haemorrhagic-stroke prevention in ageing populations cannot rely solely on antihypertensive stewardship. Aggressive abatement of fine-particulate, gaseous, acoustic, and thermal exposures implemented through equitable urban and energy policies that reshape adverse social, political and commercial determinants of health and are complemented by behaviour-supportive environments offers a pragmatic pathway to blunt the looming rise in environmentally mediated cerebral haemorrhage. Rigorous interdisciplinary research that quantifies intervention effectiveness across heterogeneous elder subgroups and explicitly incorporates spatial–temporal analytic methods will be pivotal in operationalising this agenda at scale.
